# The Influence of Short-Term Isothermal Annealing on Changes in the Surface and the Physical and Chemical Properties of Amorphous Metal Alloy Al_87_Y_4_Gd_1_Ni_4_Fe_4_

**DOI:** 10.3390/ma19204383

**Published:** 2026-10-19

**Authors:** Khrystyna Khrushchyk, Krzysztof Aniołek, Julian Kubisztal, Katarzyna Balin, Vasyl Kordan, Małgorzata Karolus, Lidiya Boichyshyn, Veronika Pihel

**Affiliations:** 1 Department of Physical and Colloidal Chemistry, Faculty of Chemistry, Ivan Franko National University of Lviv, 6 Kyryla and Mefodiia Street, 79005 Lviv, Ukraine; khrystyna.khrushchyk@us.edu.pl (K.K.); lidiya.boichyshyn@lnu.edu.ua (L.B.); veronika.pihel@lnu.edu.ua (V.P.); 2 Faculty of Science and Technology, Institute of Materials Engineering, University of Silesia in Katowice, 1A Pułku Piechoty Street, 41500 Chorzów, Poland; krzysztof.aniolek@us.edu.pl (K.A.); julian.kubisztal@us.edu.pl (J.K.); katarzyna.balin@us.edu.pl (K.B.); 3 Department of Inorganic Chemistry, Faculty of Chemistry, Ivan Franko National University of Lviv, 6 Kyryla and Mefodiia Street, 79005 Lviv, Ukraine; vasyl.kordan@lnu.edu.ua

**Keywords:** amorphous metal alloy (AMA) based on aluminum, crystallization, oxidation, electrochemical properties, tribological properties

## Abstract

This study investigates the effect of short-term isothermal annealing at 647 ± 1 K for 5 min on the surface state, electrochemical response, and tribological behavior of the Al_87_Y_4_Gd_1_Ni_4_Fe_4_ amorphous alloy. Scanning Kelvin probe measurements revealed a pronounced shift in the contact potential difference and a more spatially coherent distribution of CPD after annealing, indicating substantial modification of the surface electronic state. XPS analysis showed that the relative contribution of oxidized Al increased from approximately 84.4% to 92.9%, whereas the contribution of non-oxide Al species decreased from approximately 15.6% to 7.1%. At the same time, the total surface oxygen concentration decreased and yttrium became enriched in the near-surface region, demonstrating a redistribution of surface chemical states rather than a simple increase in oxidation. Annealing caused only minor changes in surface roughness but markedly improved wear performance: the specific wear rate decreased from 1.917 × 10^−3^ to 1.234 × 10^−3^ mm^3^/(N·m) (approximately 36%), while the maximum wear-track depth decreased from 35.8 to 8.55 μm. In contrast, electrochemical impedance spectroscopy revealed deterioration of the protective properties of the surface layer. The passive-film resistance decreased from 7845 to 4335 Ω·cm^2^, the charge-transfer resistance from 8692 to 5262 Ω·cm^2^, and the double-layer CPE exponent ndl from 0.91 to 0.74, indicating increased electrochemical heterogeneity of the interface. Thus, short-term annealing produces a distinct functional trade-off: it substantially improves the tribological performance of the Al_87_Y_4_Gd_1_Ni_4_Fe_4_ alloy while simultaneously reducing the effectiveness of its electrochemical surface barrier.

## 1. Introduction

Amorphous metal alloys (AMAs), or metallic glasses, are characterized by the absence of long-range atomic order, which gives rise to a specific set of physical and chemical, mechanical, and electrochemical properties. Unlike crystalline materials, amorphous alloys lack a regular crystal lattice, grains, and conventional grain boundaries; this promotes the formation of a homogeneous structure and reduces the number of local micro-galvanic couples. The combination of high strength, hardness, wear resistance, and corrosion resistance has generated significant interest in the application of AMAs across various fields of engineering [1,2]. Amorphous aluminum alloys alloyed with transition metals and rare-earth elements occupy a special place among them. Al–TM–RE systems are characterized by low density, high strength, and a significant capacity to form amorphous and nanocrystalline states [3,4]. It has also been shown that the annealing atmosphere significantly affects the crystallization and corrosion resistance of the amorphous Al_86_Ni_9_La_5_ alloy [5]. The amorphous state is thermodynamically metastable; therefore, under the influence of temperature, the structure of metallic glass gradually evolves toward a reduction in excess free energy. The stability of the amorphous structure is determined by a combination of factors, among which the difference in atomic radii of the components, the nature of interatomic interaction, and the negative heat of mixing are important [3,4]. In Al-containing systems, these factors contribute to the suppression of crystallization. However, upon heating, they can determine the sequence of new phase formation. At the same time, the surface properties of amorphous metallic alloys depend not only on the nominal chemical composition but also on local atomic ordering, structural heterogeneity, and the nature of interatomic bonds. Studies of the corrosion behavior of metallic glasses indicate that local structure and interatomic bond energy can be significant factors determining their resistance to corrosion-induced degradation [6]. Alloying also influences surface properties. Specifically, in Al–Cu–Ni–Y systems, the addition of copper promotes the formation of a denser structure and can increase the alloy’s polarization resistance [7]. For aluminum alloys alloyed with Fe and Y, varying the component ratio affects both the glass-forming ability and the surface corrosion resistance [8]. Micro-alloying with Ti or Cr in amorphous Al–Ni–Y alloys also significantly affects their corrosion behavior [9]. At the same time, the use of powder metallurgy techniques enables the production of bulk Al–Ni–La alloys while preserving the advantages of an amorphous or nanocrystalline structure and ensuring high mechanical properties [10]. The formation of a nanocrystalline structure in Al-based alloys can also contribute to a simultaneous increase in hardness and corrosion resistance, particularly through the formation of finely dispersed crystalline phases [11]. The first stage of the structural evolution of amorphous metallic alloys (AMAs) during heating is typically structural relaxation. This process occurs at temperatures below the crystallization temperature and is accompanied by a rearrangement of the local atomic environment, a reduction in excess structural energy, and densification of the atomic packing. A key parameter in this process is the free volume, which arises from the rapid cooling of the melt and the deviation of the structure from its equilibrium state [12,13]. Upon heating, a portion of the excess free volume annihilates, leading to structural densification and changes in physical properties. A similar effect of sub-Tg annealing on corrosion resistance has also been observed in Cu–Zr amorphous alloys [14]. Structural relaxation is a complex process involving the rearrangement of local atomic configurations and changes in the number of structural regions capable of undergoing local shear transformations. Studies of microscopic relaxation mechanisms indicate that changes in the structural state of metallic glasses are linked to the dynamics of shear transformation zones, which play a crucial role in determining the material’s plasticity and mechanical stability [15]. However, excessive relaxation can reduce the amorphous structure’s capacity for further plastic rearrangement and contribute to embrittlement [16]. In general, the thermal evolution of metallic glasses is determined by the competition between structural densification, the relief of internal stresses, and subsequent phase decomposition [17]. These general patterns of crystallization in metallic glasses and supercooled liquids are consistent with the findings of review studies [18]. An important consequence of structural relaxation is the alteration of diffusion conditions for alloying components. During heating, their redistribution within near-surface layers can occur, directly influencing the formation of the passive film. For amorphous Al–Ni–Y alloys, it has been shown that low-temperature annealing promotes yttrium segregation to the surface, which can enhance passivation and corrosion resistance [19]. Similar processes of alloying element redistribution are observed in Fe-based amorphous alloys, where heat treatment can promote the densification of the passive film, whereas the subsequent formation of crystalline phases leads to the local depletion of specific components [20]. Structural relaxation processes are directly linked to the subsequent nucleation of crystalline phases. Changes in local structure and free energy can influence the energy barrier for crystal nucleus formation [21].

In this context, regions of local chemical inhomogeneity may form within the amorphous matrix, creating favorable conditions for subsequent nanocrystallization [22]. Thus, structural relaxation and crystallization are not always entirely distinct processes but can constitute a sequential structural evolution. As the heat treatment temperature increases, devitrification of the amorphous structure occurs. The general principles governing crystallization in metallic glasses and supercooled liquids are summarized in the review literature [18]. Primary crystallization, resulting in the formation of α-Al nanocrystals, is characteristic of Al-based amorphous metallic alloys. The formation of such nanocrystals can occur with a high nucleation density and be accompanied by the development of a nanocrystalline structure within the amorphous matrix [23]. In Al–La–Ni systems, crystallization proceeds in multiple stages: the first stage involves the precipitation of fcc-Al, whereas further heating promotes the formation of intermetallic phases [24]. Such processes can be accompanied by a significant increase in material hardness due to the formation of a nanocrystalline structure [16]. The kinetics and sequence of crystallization are determined by the composition of the amorphous alloy. In Al-based systems, the atomic sizes of the components and the nature of their interactions play a crucial role, influencing the stability of the amorphous phase and the subsequent mechanism of its decomposition [25]. In situ X-ray diffraction studies demonstrate that altering the alloy composition—specifically by substituting Ni or introducing Fe and Cu—can significantly modify the set of phases formed during heating [26]. This creates opportunities to control the surface structure through targeted alloying. At the same time, crystallization does not always have a negative impact on the functional properties of the surface. For instance, short-term annealing of Al_87_Y_4_Gd_1_Ni_4_Fe_4_ at 647 ± 1 K promotes the formation of AlFe_2_Ni and GdFe_2_ nanodomains, leading to a significant increase in the alloy’s electrocatalytic activity in the hydrogen evolution reaction [27]. In some cases, the formation of a nanocrystalline structure can also contribute to enhanced corrosion resistance. For Al–Ni–Y alloys, it has been shown that moderate structural relaxation and the formation of fine nanocrystals can improve surface protective properties, whereas an increase in the size of crystalline regions promotes the formation of local galvanic couples and impairs corrosion resistance [2]. A similar dependence of corrosion resistance on crystallization has been demonstrated for the Cu_50_Zr_45_Al_5_ bulk amorphous alloy [28]. In Al–Ni–La–Cu systems, the fully amorphous state is characterized by high corrosion resistance, whereas subsequent crystallization can lead to its reduction [29]. Conversely, for certain Al–Ni–La alloys, partial crystallization can result in the formation of a nanocomposite structure with enhanced corrosion resistance [30]. In certain Al–Ni–La–Zr systems, primary crystallization can temporarily improve corrosion properties, whereas the subsequent formation of secondary intermetallic phases leads to their deterioration [31]. For Al–Ni–Zr alloys, changes in the oxide film composition—particularly resulting from the formation of the nanocrystalline α-Al phase—also play a significant role in this process [32]. Oxidation is one of the most important mechanisms altering the surface of amorphous metal alloys (AMAs) during heat treatment. Oxidation processes can modify the composition and structure of the surface layer, influence the onset temperature of crystallization, and promote surface crystallization [33]. Oxygen can facilitate the selective oxidation of specific components of the amorphous alloy, thereby creating conditions for the nucleation of crystalline phases directly at the surface [34]. Consequently, the heat treatment atmosphere is a crucial parameter determining the final structure and properties of the surface layer. The formation of the passive film largely determines the corrosion behavior of amorphous metallic alloys (AMAs). For Al–Ni–Ce alloys, it has been established that surface hydroxide compounds play a crucial role in the stability of the passive layer [35]. At the same time, alloying with rare-earth elements can have both positive and negative effects. Specifically, La can enhance the stability of the passive film, whereas the formation of certain Ce compounds may contribute to the appearance of defects in the protective layer [36]. Similarly, for Mg-based AMAs, it has been shown that the composition of the surface oxide layer is determined by the ratio of alloying components, and the formation of multicomponent oxides is a key factor in passivation [37]. The transition from the amorphous to the crystalline state also significantly alters the corrosion behavior of the Mg_67_Zn_28_Ca_5_ alloy [38]. Controlled oxidation can be employed as a specialized surface modification method. For Fe-based amorphous alloys, pre-oxidation promotes the diffusion of passivating elements toward the oxidation front and the formation of a more protective surface layer [39]. At the same time, heat treatment of amorphous coatings at excessively high temperatures can lead to the formation of micropores. Despite a potential reduction in surface roughness, the emergence of porosity negatively affects the coating’s protective properties [40]. The nature of the surface morphology following short-term heat treatment is also of significant importance. For Al_87_Y_4_Gd_1_Ni_8_, it has been established that brief annealing can promote densification of the surface layer and enhance its functional properties, whereas extending the heat treatment duration leads to increased roughness and a deterioration of surface characteristics [1]. Thus, the heat treatment time is a parameter no less critical than temperature; even at a constant temperature, prolonging the holding time can shift the material from beneficial structural relaxation to intense crystallization, segregation, and degradation of the surface layer. It should be noted that the patterns established for different classes of metallic glasses are not always identical. For Cu-based amorphous alloys, partial crystallization can in some cases enhance corrosion resistance, whereas complete crystallization impairs protective properties [28]. For Fe-based amorphous alloys, heat treatment can also lead to either an improvement or a deterioration in corrosion behavior, depending on the temperature, holding time, and the nature of the phases formed [20,40]. Furthermore, the melt temperature during fabrication additionally influences the subsequent corrosion behavior of Fe-based amorphous alloys [41]. Complete crystallization of Fe–Ni–B–Si alloys, resulting in the formation of the γ-phase and borides, typically promotes chemical inhomogeneity and impairs corrosion resistance [42]. Thus, surface changes in amorphous metallic alloys during heat treatment are determined by the interplay of several interconnected processes: structural relaxation, free volume reduction, diffusional redistribution of components, oxidation, passive film formation, and crystallization. The chemical state of the surface and the composition of oxide compounds formed during the material’s interaction with the environment also play a significant role in corrosion behavior [35].

For this reason, heat treatment can serve as a tool for the targeted control of the morphology, phase composition, passivity, and surface functional properties of amorphous metal alloys, which aligns with recent reviews on the corrosion behavior of metallic glasses and their composites [43].

Recent studies indicate that the mechanical and electrochemical responses of Al-based amorphous materials to annealing do not necessarily evolve in parallel. For Al_84_Ni_9_Y_7_ metallic glass, the highest hardness and the most favorable electrochemical response were observed under different thermal conditions, indicating that mechanical strengthening does not necessarily coincide with improved corrosion protection [44]. Annealing-induced devitrification of AlFeSi metallic-glass coatings was shown to improve dry-sliding wear resistance, whereas the current density induced by sliding in 3.5 wt.% NaCl increased with annealing temperature [45]. Complementary electrochemical studies of AlFeSi metallic-glass coatings demonstrated that progressive devitrification, nanocrystal growth, and increasing structural heterogeneity increase the corrosion current density and reduce impedance [46]. Recent tribocorrosion studies on Al-based amorphous coatings further demonstrate that phase structure, hardness, corrosion resistance, and wear behavior are closely interrelated under chloride-containing conditions [47]. Thus, a heat-treatment condition favorable for wear resistance need not maximize electrochemical protection. In the present work, however, tribological and electrochemical responses were measured independently; therefore, the term functional trade-off refers to their opposite trends and does not imply a simultaneous tribocorrosion test.

In our previous work [1], short-term annealing of the Al_87_Y_4_Gd_1_Ni_8_ amorphous alloy at 611 ± 1 K for 5 min was shown to modify the surface layer and improve its electrochemical and tribological properties. It is equally important to investigate whether the trend toward improved electrochemical and tribological properties is maintained in the alloy alloyed with 4 at.% iron. Furthermore, it is necessary to assess the risks and the impact of alloying with 4 at.% iron on electrochemical behavior in a 0.3% aqueous NaCl solution. Additionally, the surfaces of the Al_87_Y_4_Gd_1_Ni_8_ and Al_87_Y_4_Gd_1_Ni_4_Fe_4_ amorphous alloys should be investigated and compared using the scanning Kelvin probe (SKP) technique following heat treatment. The present study focuses on the Fe-containing Al_87_Y_4_Gd_1_Ni_4_Fe_4_ alloy. The annealing temperature of 647 ± 1 K corresponds to the second phase transition previously identified from DSC analysis at a heating rate of β = 20 K min^−1^ [1]. The aim of this study was to elucidate the effect of short-term isothermal annealing at 647 ± 1 K for 5 min on the surface state and functional properties of the Al_87_Y_4_Gd_1_Ni_4_Fe_4_ amorphous alloy, with particular emphasis on the relationship between changes in surface chemical composition, oxide-film heterogeneity, and its electrochemical and tribological behavior.

## 2. Materials

The object of the investigations was the AMA Al_87_Y_4_Gd_1_Ni_4_Fe_4_ in the form of ribbons with a thickness and width of 20–25 μm and 3 mm, respectively, which were obtained at the G. V. Kurdyumov Institute for Metal Physics of the Ukrainian Academy of Sciences (Kyiv) by a melt-spinning method in a helium atmosphere on a copper drum rotating at a speed of ~30 m/s. Melt quenching from the liquid state was carried out at a rate of 10^6^ K/s in a helium atmosphere. Due to the specific technology used to produce amorphous metal ribbons from melts, a distinction is made between the contact side—which is in direct contact with the cooling element—and the outer side of the ribbon. In this study, experiments were conducted for the contact side of the alloy. The melt was prepared from pure metals and the binary compound REAl_3_ (RE = Y, Gd). The purity of the starting metals was as follows: Al (99.999 wt.%), Ni (99.99 wt.%), Y (99.96 wt.%), Gd (99.96 wt.%) and Fe (99.99 wt.%).

## 3. Methods of Investigation

### 3.1. Scanning Kelvin Probe (SKP) Method

The surface electronic properties of the tested materials were evaluated based on contact potential difference (CPD) maps recorded in air using the scanning Kelvin probe (SKP) technique. The SKP measurements enabled the determination of the spatial distribution of CPD on the sample surfaces after thermal treatment.

The CPD maps were recorded using a PAR Model 370 scanning electrochemical workstation (Princeton Applied Research, Oak Ridge, TN, USA) equipped with a tungsten Kelvin probe (Princeton Applied Research, Oak Ridge, TN, USA). The scanned area was 3000 × 3000 µm. The measurements were performed in air at 22 °C and at a relative humidity of 25%. The probe-to-sample distance was set to approximately 100 µm.

The *CPD* maps were subjected to statistical analysis to determine the arithmetic mean (*CPD*_av_), root mean square deviation (*CPD*_rms_), skewness (*CPD*_sk_), excess kurtosis (*CPD*_ku_), and autocorrelation length (*CPD*_al_). The *CPD*_rms_ parameter quantifies the magnitude of local *CPD* fluctuations relative to the arithmetic mean, whereas *CPD*_sk_ describes the asymmetry of the *CPD* distribution, and *CPD*_ku_ characterizes its peakedness and tail weight. For an ideal Gaussian distribution, both skewness and excess kurtosis are equal to zero. The *CPD*_al_ parameter was determined as the lateral distance at which the two-dimensional autocorrelation function decays to 1/e of its zero-lag value. It therefore describes the distance over which *CPD* values remain spatially correlated on the material surface.

### 3.2. Surface Roughness

Surface roughness and wear mark geometry tests following tribological testing were performed using a Mitutoyo Surftest SJ-500 2D contact profilometer (Mitutoyo, Tokyo, Japan). Roughness measurements of the samples were performed in the longitudinal direction. Roughness was measured in accordance with ISO 21920 using an elementary section and a measuring section selected according to the surface roughness [48].

### 3.3. Tribological Characteristics

Tribological characteristics were tested using a TRN Tribometer operating in a ball-on-disc system (Anton Paar, Corcelles-Cormondrèche, Switzerland). The tribological tests were performed in a linear reciprocating motion in dry contact. The test parameters were selected on the basis of preliminary tests. The tests were performed at a load of 1 N with a frequency of 5 Hz over a friction distance of 100 m. The amplitude of movement was 10 mm. The maximum linear speed was 15.71 cm/s. The tests were carried out at a temperature of 21 ± 1 °C and a humidity of 40 ± 5%. Balls with a diameter of 6 mm made of zirconium oxide (ZrO_2_) were used as counter-samples. After the tribological tests, the average surface area of the wear marks was evaluated using a Surftest SJ-500 contact profilometer (Mitutoyo, Tokyo, Japan). The specific wear rate was calculated as k = V/(F·L), where V is the wear volume determined from the profilometric wear-track geometry, F is the applied normal load, and L is the total sliding distance.

### 3.4. X-Ray Photoelectron Spectroscopy (XPS) Method

The elemental composition of the investigated samples was determined using X-ray photoelectron spectroscopy (XPS). The analyses were performed with a PHI 5700 system from Physical Electronics (Chanhassen, MN, USA), utilizing a monochromatic Al Kα radiation source (1486.6 eV). Spectral data were recorded with pass energies set to 187.85 eV for survey scans and 23.50 eV for high-resolution core-level spectra. A flood gun was used to neutralize charge buildup caused by X-ray irradiation. Subsequent quantitative evaluation and curve-fitting procedures were conducted in the MULTIPAK environment (version 9.6.0.1, ULVAC PHI, Chigasaki, Japan). The energy scale was subsequently corrected by aligning the adventitious carbon C1s signal to 284.8 eV. A Shirley background was subtracted from all core-level spectra prior to fitting with Gaussian–Lorentzian sum functions.

### 3.5. Electrochemistry Measurement

Electrochemical measurements were performed for the as-cast and annealed Al_87_Y_4_Gd_1_Ni_4_Fe_4_ alloys in 0.3% NaCl at 293 K using a PARSTAT 2273 potentiostat (Princeton Applied Research, AMETEK, Berwyn, PA, USA) in a three-electrode cell. The alloy (0.5 cm^2^) served as the working electrode, a saturated calomel electrode (SCE, saturated KCl, E = 0.242 V vs. SHE) as the reference electrode, and Pt as the counter electrode. OCP was recorded until stabilization (−0.594 V for the as-cast alloy and −0.643 V after annealing). Potentiodynamic polarization curves were recorded in the same electrolyte over the potential range from −1.5 to −0.1 V vs. SCE at a scan rate of 50 mV s^−1^, and E_corr and i_corr were determined by Tafel analysis. For each sample state, three independent experimental series were performed (n = 3). Within each series, five consecutive potentiodynamic polarization cycles were recorded. For each cycle number, the E_corr and i_corr values obtained in the three independent series were averaged; thus, the five consecutive cycles were treated as repeated measurements within each experimental series and not as independent replicates. The relative SD did not exceed 2%. EIS spectra were collected at OCP from 20 kHz to 10 mHz using a 10 mV perturbation. The spectra were fitted with an equivalent circuit containing R_s, R_f, R_ct, CPE_f, and CPE_dl; the CPE exponents n_f and n_dl were used to characterize deviations from ideal capacitive behavior.

### 3.6. Surface Morphology and Elemental Analysis

The surface morphology was studied using scanning electron microscopy (SEM) (Tescan Brno s.r.o. Tescan Vega 3 LMU, 623 00 Brno, Czech Republic). The chemical composition and distribution of elements were determined by energy-dispersive spectroscopy (EDS), including mapping of the elements O, Al, Fe, Ni, Y, and Gd. The analysis was performed for the initial and annealed samples before and after tribological testing.

## 4. Results and Discussion

### 4.1. Scanning Kelvin Probe (SKP) Analysis of the Surfaces of Al_87_Y_4_Gd_1_Ni_8_ and Al_87_Y_4_Gd_1_Ni_4_Fe_4_ Amorphous Alloys in the As-Cast State and After Annealing for 5 min at T_3_ = 611 ± 1 K and 647 ± 1 K, Respectively

In our previous article [1], it was established that the electrochemical properties of the Al_87_Y_4_Gd_1_Ni_8_ amorphous metallic alloy (AMA) improve upon heat treatment at 611 ± 1 K for 5 min. Annealing temperatures (647 ± 1 K) for the Al_87_Y_4_Gd_1_Ni_4_Fe_4_ AMA were determined from DSC curves [30].

Using XPS method, stable aluminum oxide passivation and progressive yttrium hydroxylation were identified as the defining characteristics of the surface chemical composition of the Al_87_Y_4_Gd_1_Ni_8_ alloy, with their kinetics governed by competing segregation and oxidation processes.

**Figure 1:**
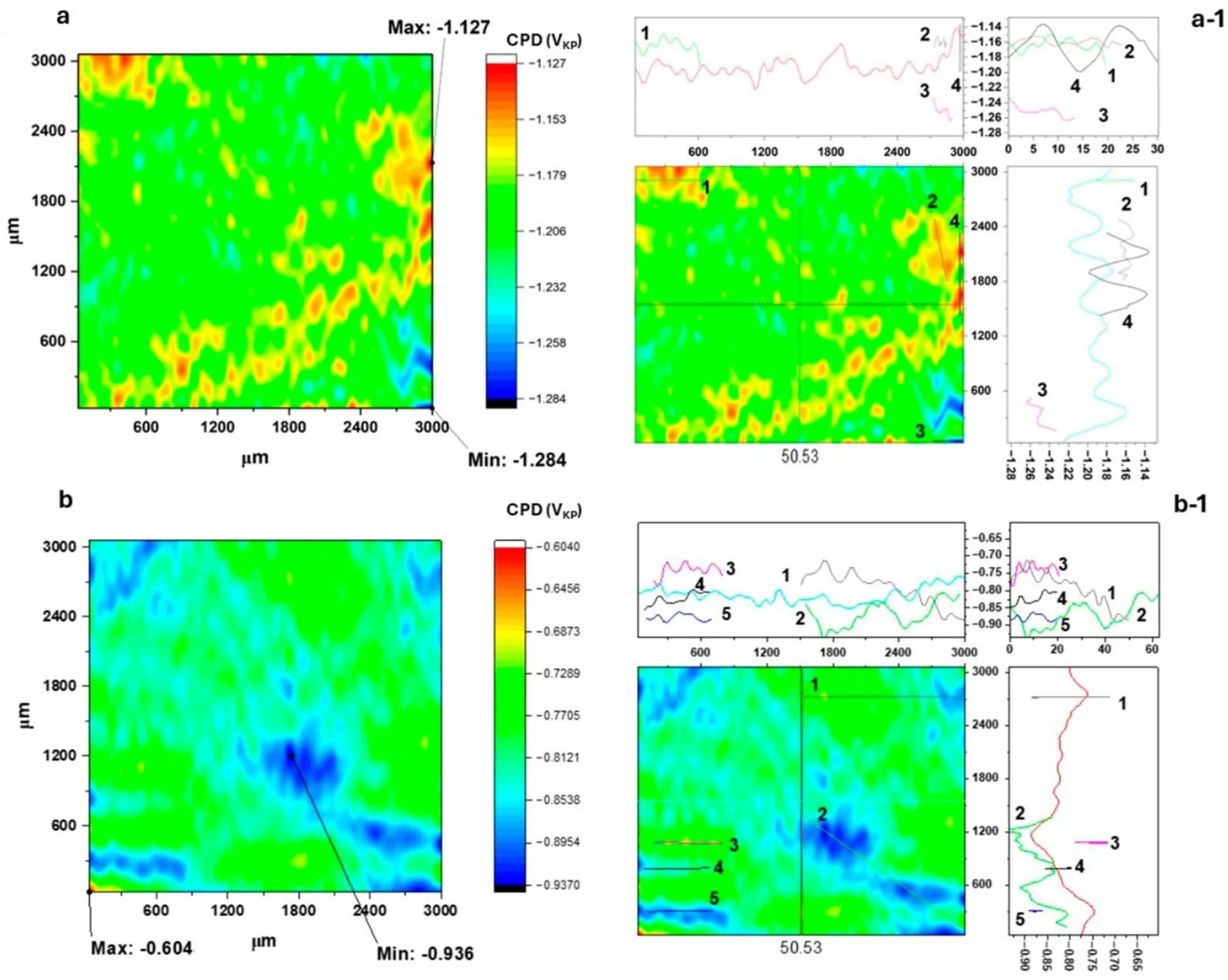
Contact potential difference maps recorded for the AMA Al_87_Y_4_Gd_1_Ni_8_ (**a**,**a-1**) initial and (**b**,**b-1**) annealed during 5 min. at T_3_ = 611 ± 1 K (contact sides of AMA).

**Figure 2:**
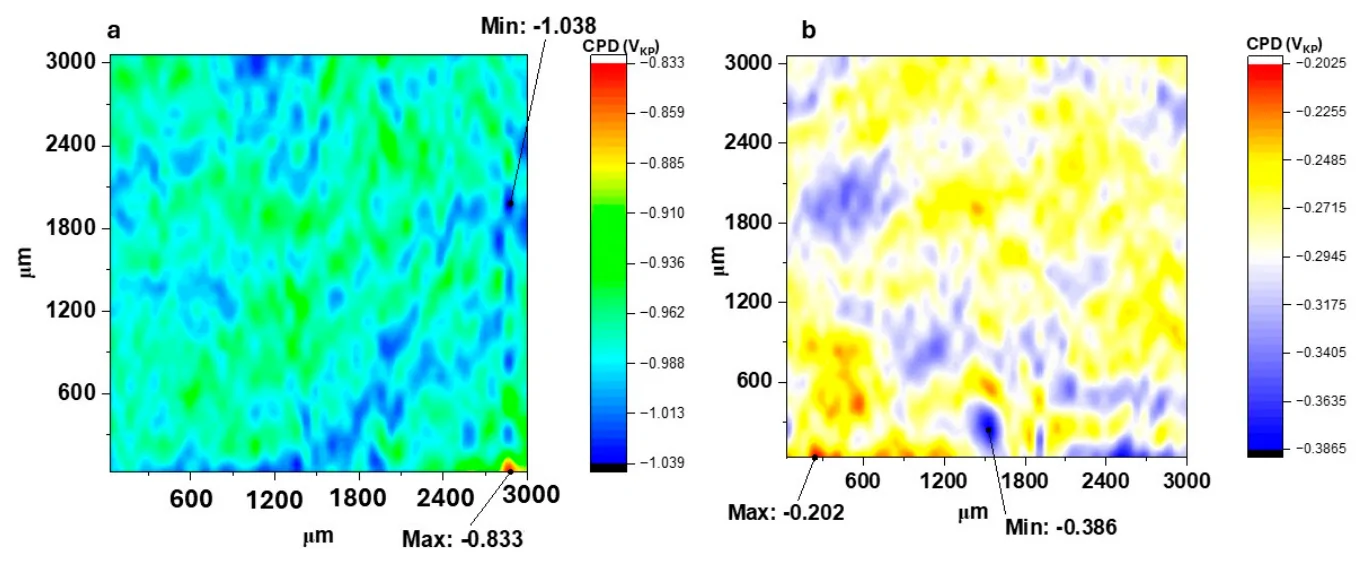
Contact potential difference maps recorded for the AMA Al_87_Y_4_Gd_1_Ni_4_Fe_4_ (**a**) initial and (**b**) annealed during 5 min. at T_3_ = 647 ± 1 K (contact sides of AMA).

Figure 1 and Figure 2 show the contact potential difference maps recorded for the Al_87_Y_4_Gd_1_Ni_8_ and Al_87_Y_4_Gd_1_Ni_4_Fe_4_ amorphous alloys in the as-cast state and after annealing. The statistical parameters calculated from the *CPD* maps are summarized in Table 1.

Annealing caused a pronounced shift in the mean *CPD* towards more positive values for both alloys. For Al_87_Y_4_Gd_1_Ni_8_, *CPD*_av_ increased from −1.193 V in the as-cast state to −0.813 V after annealing at 611 ± 1 K, corresponding to a shift of approximately 0.380 V (Figure 1a,b). An even larger shift was observed for Al_87_Y_4_Gd_1_Ni_4_Fe_4_, for which *CPD*_av_ increased from −0.978 to −0.285 V after annealing at 647 ± 1 K (Figure 2a,b). These changes indicate a substantial modification of the surface electronic state induced by heat treatment. In the case of Al_87_Y_4_Gd_1_Ni_8_, this interpretation is consistent with the previously reported annealing-induced changes in the chemical state of the surface oxide layer [1]. The XPS results obtained for Al_87_Y_4_Gd_1_Ni_4_Fe_4_ also indicate a redistribution of surface chemical states after annealing.

The amplitude of the local *CPD* variations responded differently in the two alloys. For Al_87_Y_4_Gd_1_Ni_8_, *CPD*_rms_ increased from 18.0 to 39.0 mV, while the total *CPD* range (calculated as *CPD*_max_—*CPD*_min_) increased from 157 to 332 mV. Both parameters therefore indicate that annealing markedly increased the amplitude of the *CPD* variations across the alloy surface. For Al_87_Y_4_Gd_1_Ni_4_Fe_4_, *CPD*_rms_ increased more moderately, from 16.3 to 21.0 mV, whereas the total *CPD* range decreased slightly from 205 to 183 mV. Consequently, the amplitude of the *CPD* variations remained of the same order of magnitude for this alloy.

Annealing clearly affected the spatial distribution of the CPD values on the surfaces of both investigated alloys. The CPDal value increased from 163.2 to 211.9 µm for Al_87_Y_4_Gd_1_Ni_8_ and from 113.6 to 271.8 µm for Al_87_Y_4_Gd_1_Ni_4_Fe_4_. The increase in autocorrelation length indicates that, after annealing, the local CPD values remained correlated over longer lateral distances. In other words, the CPD maps contained larger regions with similar CPD values. For Al_87_Y_4_Gd_1_Ni_8_, the simultaneous increases in CPDal and CPDrms indicate the formation of larger CPD domains accompanied by greater potential contrast. For Al_87_Y_4_Gd_1_Ni_4_Fe_4_, the pronounced increase in CPDal, together with the comparatively small change in CPDrms and the slight reduction in the total CPD range, suggests a more spatially coherent distribution of the surface potential after annealing.

The different evolution of CPD_rms and CPD_al in the two alloys can be related to their different transition-metal compositions and, consequently, to different structural evolution during short-term annealing. For Al_87_Y_4_Gd_1_Ni_4_Fe_4_, annealing under the same conditions was previously shown to induce the formation of AlFe_2_Ni- and GdFe_2_-containing nanophases [27]. Such subsurface phase formation and the associated redistribution of alloying elements may modify the local electronic environment and promote the development of larger laterally coherent surface-potential domains. This interpretation is consistent with the pronounced increase in CPD_al accompanied by only a moderate increase in CPD_rms. In contrast, the stronger increase in CPD_rms observed for Al_87_Y_4_Gd_1_Ni_8_ indicates a greater annealing-induced local potential contrast. Since XPS does not detect Fe in the outermost surface layer, its influence is interpreted as indirect, through Fe-containing subsurface nanophases and associated structural and chemical redistribution rather than direct Fe surface segregation. It should also be noted that the two alloys differ simultaneously in Fe and Ni contents; therefore, the present comparison does not isolate the effect of Fe alone.

The skewness and excess kurtosis provide additional information about the shape of the CPD distributions. The distribution obtained for Al_87_Y_4_Gd_1_Ni_8_ after annealing was the closest to a Gaussian distribution, as indicated by its skewness and excess kurtosis values reported in Table 1. The CPD distribution for as-cast Al_87_Y_4_Gd_1_Ni_8_ exhibited modest negative skewness and positive excess kurtosis, indicating a moderate departure from an ideal Gaussian distribution. The strongest departure was observed for the as-cast Al_87_Y_4_Gd_1_Ni_4_Fe_4_, for which the positive skewness of 0.470 and excess kurtosis of 3.750 indicate an asymmetric distribution with a greater contribution of extreme CPD values than expected for a Gaussian distribution. After annealing, the distribution for this alloy became negatively skewed (CPDsk = −0.448), while the positive excess kurtosis remained appreciable (CPDku = 1.107). Therefore, based on these statistical descriptors, only the CPD distribution obtained for Al_87_Y_4_Gd_1_Ni_8_ after annealing can be regarded as approximately Gaussian.

### 4.2. X-Ray Photoelectron Spectroscopy (XPS) Analysis of the Surfaces of AMA Al_87_Y_4_Gd_1_Ni_4_Fe_4_ Amorphous Alloys in the As-Cast State and After Annealing for 5 min at T_3_ = 647 ± 1 K

The surface chemical composition of the samples examined by XPS confirmed the presence of aluminum, yttrium, oxygen, carbon, and trace amounts of nitrogen and silicon. On the surface region Gd, Fe and Ni were not detected. Quantitative XPS analysis revealed the surface atomic concentrations of the main constituents, which are summarized in Table 2. Carbon is the dominant contaminant on both samples, originating from unavoidable atmospheric exposure. As it primarily reflects surface handling rather than the material itself, the discussion will focus on the relative changes in the O/Al, O/Y, and Al/Y atomic ratios.

**Figure 3:**
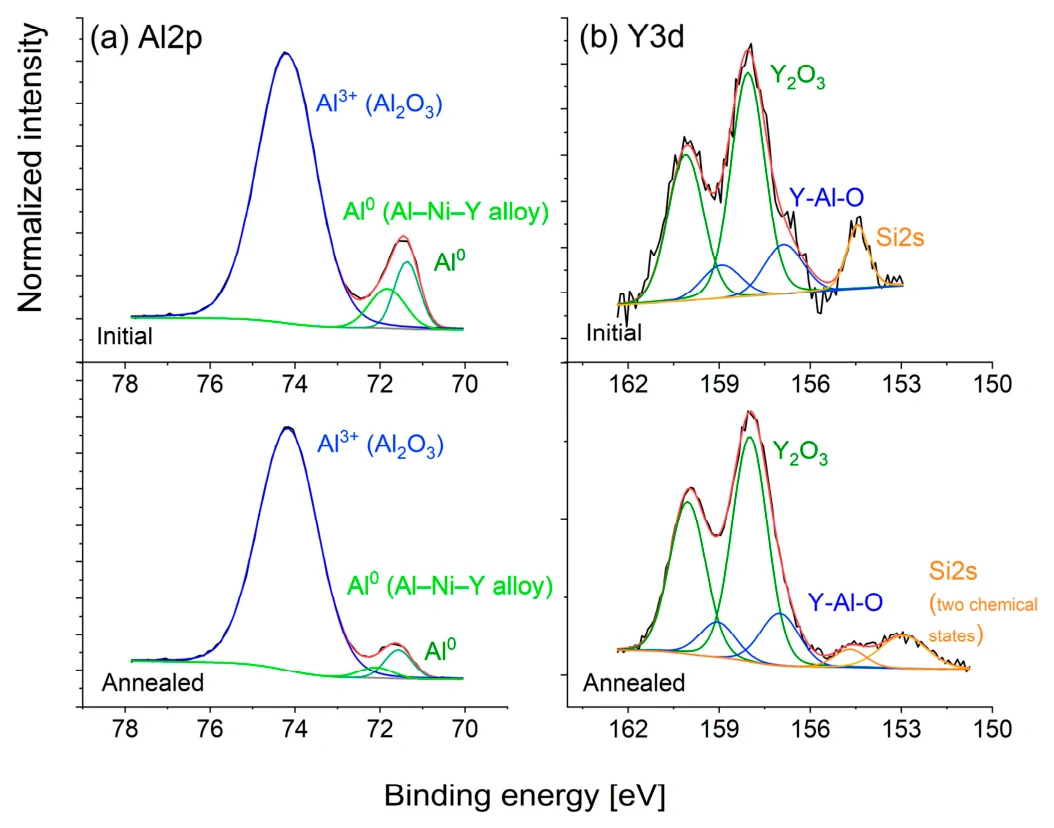
Deconvolution of high-resolution XPS spectra of (**a**) Al2p and (**b**) Y3d for samples before and after annealing. The black lines represent the experimental data, the gray lines denote the background, and the red lines correspond to the composite spectra. The other colored lines (e.g., blue, green, etc.) are attributed to the specific chemical states of the respective elements.

The aluminum surface concentration remains stable after annealing at ~16.6–16.8 at.%. In contrast, oxygen decreases from 38.6 to 33.0 at.%, reducing the O/Al ratio from 2.30 to 1.99. Although still above the stoichiometric value for Al_2_O_3_ (1.50), indicating an oxygen-rich surface, the lower ratio suggests partial removal of excess oxygen during annealing. Yttrium shows different behaviour; its surface concentration increases from 0.36 to 0.53 at.%, indicating thermally driven surface segregation. Accordingly, the Al/Y ratio decreases from 46.6 to 31.3, confirming yttrium enrichment in the near-surface region. The O/Y ratio also drops markedly, suggesting that the yttrium-enriched surface becomes relatively less oxygen-rich per Y atom. Together with high-resolution XPS data (Figure 3), these trends indicate the formation of an aluminum-dominated oxide layer significantly enriched in yttrium after heat treatment.

High-resolution Al 2p and Y 3d core-level spectra (Figure 3a,b, Table 3) were analyzed in detail to characterize the chemical states of the primary metallic constituents. The deconvoluted Al 2p core-level spectra revealed a more complex structure than in our previous studies [1], requiring three components for an adequate fit in both the initial and annealed samples (Figure 3a). The dominant feature in both cases is the peak at ~74.2 eV, attributed to fully oxidized aluminum (Al^3+^), characteristic of a surface Al_2_O_3_ layer [49]. The two remaining components appear at lower binding energies, characteristic of non-oxidized aluminum. The lower-energy state at 71.4–71.6 eV corresponds to reported values for pure Al (71.5 eV) [50], while the higher-energy state at 71.8–72.1 eV is attributed to aluminum in the alloy matrix, consistent with Al–Ni–Y alloys (72.0 eV) [51]. Both are below the standard reference for metallic Al (72.6 eV) [49]. Its relative contribution increases from ~84.4% in the as-cast sample to ~92.9% after annealing, indicating that a larger fraction of the surface aluminum is present in the oxidized state after heat treatment. In contrast, the combined contribution of the two non-oxide Al components decreases from ~15.6% to ~7.1%. This indicates a substantial decrease in the fraction of metallic or alloyed aluminum exposed within the XPS information depth. Together with the survey XPS data, these results point to a redistribution of the surface chemical states rather than a simple increase in the total oxygen content.

The high-resolution Y 3d spectra (Figure 3b) were deconvoluted into two spin–orbit split doublets (Y 3d_5/2_ and Y 3d_3/2_), indicating the presence of two distinct chemical environments for yttrium in both samples. Although the relative proportions of these two states remain remarkably stable upon annealing (~60% and ~40%, respectively), a clear shift in their binding energies is observed. In the initial sample, the first doublet, with Y 3d_5/2_ at 156.9 eV, is assigned to yttrium oxide (Y_2_O_3_ or a sub-stoichiometric oxide) [1,52]. The second Y 3d_5/2_ component, located at 158.9–159.1 eV, is assigned to yttrium in a mixed Y–O–Al chemical environment, suggesting incorporation of yttrium into the Al-rich surface oxide layer. After annealing, both Y 3d components shift slightly to higher binding energy, from 156.9 to 157.0 eV for the Y_2_O_3_-related component and from 158.9 to 159.1 eV for the Y–O–Al component. The nearly unchanged relative contribution of the two components (~60% and ~40%) indicates that the local yttrium coordination remains comparatively stable, whereas the small binding-energy shift reflects a modification of the surrounding chemical environment rather than a phase transformation. Together with the SKP results, the XPS data indicate that short-term annealing increases the relative contribution of oxidized Al and modifies the spatial and chemical state of the Al-rich surface layer.

### 4.3. Investigation of Changes in the Surface Profile and Tribological Properties of AMA Al_87_Y_4_Gd_1_Ni_4_Fe_4_ Following Annealing for 5 min at a Temperature of T = 647 ± 1 K

**Figure 4:**
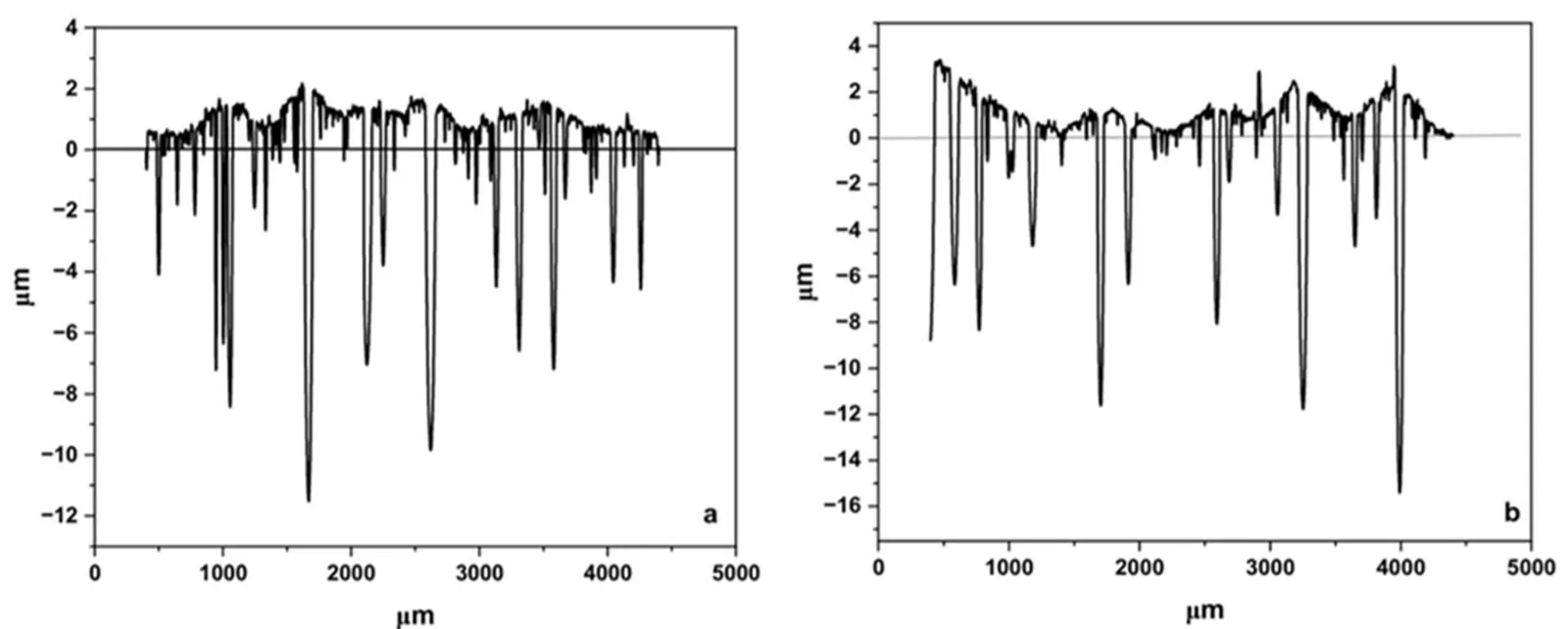
AMAs Al_87_Y_4_Gd_1_Ni_4_Fe_4_ surface profiles of the contact side (width × height): (**a**) as-cast and (**b**) annealed for 5min at T = 647 ± 1 K.

Analysis of the surface profile (Figure 4) of the amorphous alloy Al_87_Y_4_Gd_1_Ni_4_Fe_4_ for the as-cast sample and the sample annealed for 5 min at 647 ± 1 K revealed no significant changes in topography, as was the case in our previous study on the alloy Al_87_Y_4_Gd_1_Ni_8_ [1]. For the Al_87_Gd_5_Ni_8_ amorphous alloy, the roughness parameters are Ra = 1.39 µm, Rq = 2.22 µm, Rz = 10.88 µm for the as-cast alloy, and Ra = 1.47 µm, Rq = 2.14 µm, Rz = 9.65 µm [53].

In its as-cast state, the surface of the Al_87_Y_4_Gd_1_Ni_4_Fe_4_ AMA is characterised by a relatively uniform profile (Table 4) with an average roughness of R_a_ = 1.29 µm, a quadratic mean of R_q_ = 1.95 µm and a maximum roughness height of R_z_ = 9.52 µm, which indicates the compactness and stability of the film. Following a short-term annealing treatment (5 min), a slight increase in all roughness parameters is observed (R_a_ = 1.33 µm, R_q_ = 2.01 µm, R_z_ = 9.92), indicating the formation of surface irregularities and microporosity.

Our previous studies have shown an improvement in the tribological properties [1,53] as a result of thermal annealing for 5 min at the temperatures of the second phase transition in the steady-state crystallisation process for the AMAs Al_87_Gd_5_Ni_8_ and Al_87_Y_4_Gd_1_Ni_8_ at 601 ± 1 and 611 ± 1 K, respectively.

**Figure 5:**
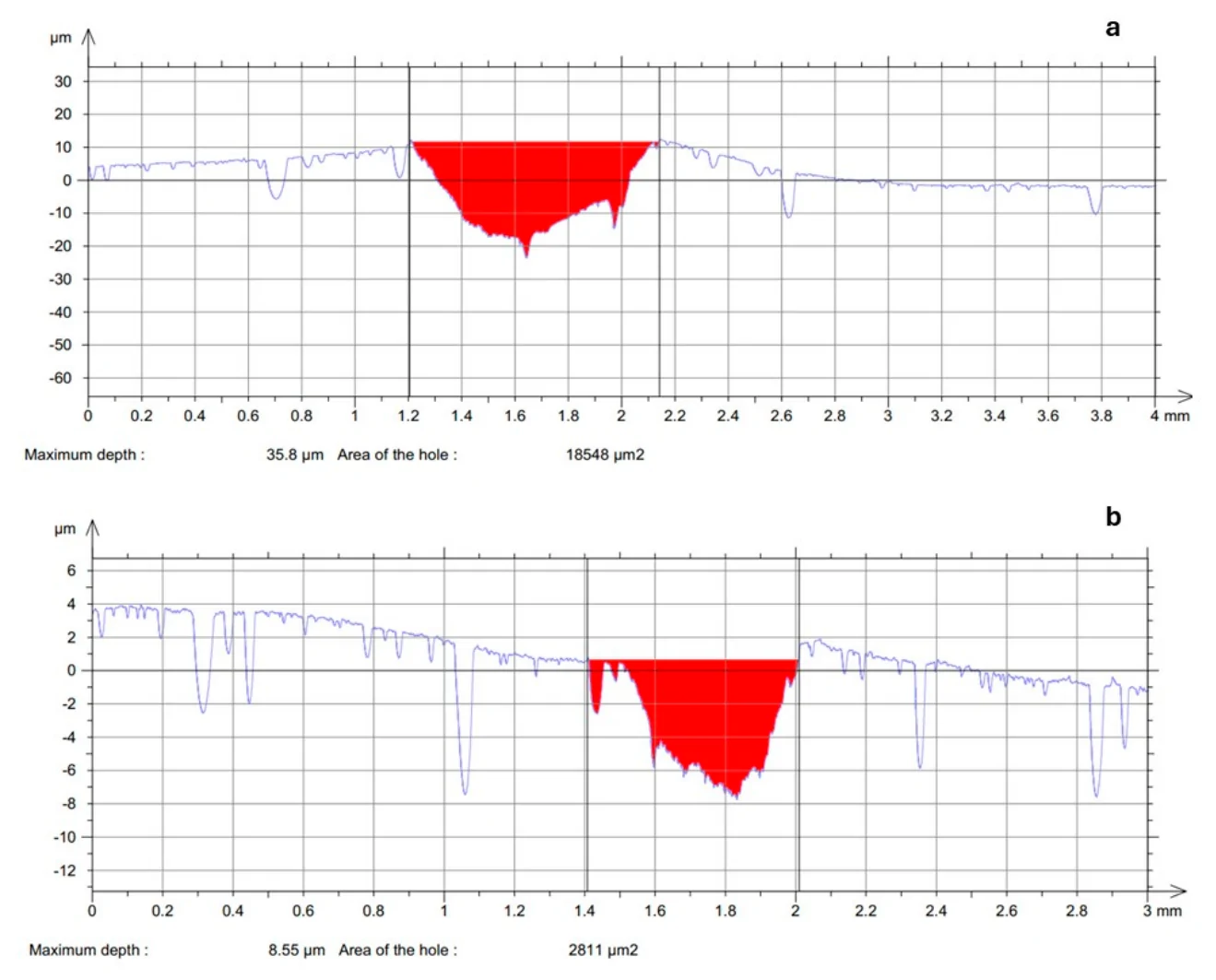
AMA surface profiles of the contact side (width × height) of Al_87_Y_4_Gd_1_Ni_4_Fe_4_ (**a**) initial and (**b**) annealed for 5 min at T_3_ = 647 ± 1 K after tribological tests (dry contact).

Quantitative wear evaluation confirmed the profilometric trend. After annealing for 5 min at 647 ± 1 K, the specific wear rate decreased from 1.917 × 10^−3^ to 1.234 × 10^−3^ mm^3^/(N·m) (approximately 36%), the wear-scar width from 973 to 728 μm, and the maximum wear-track depth from 35.8 to 8.55 μm. Thus, short-term annealing improves wear performance under the applied dry-sliding conditions (Figure 5).

**Figure 6:**
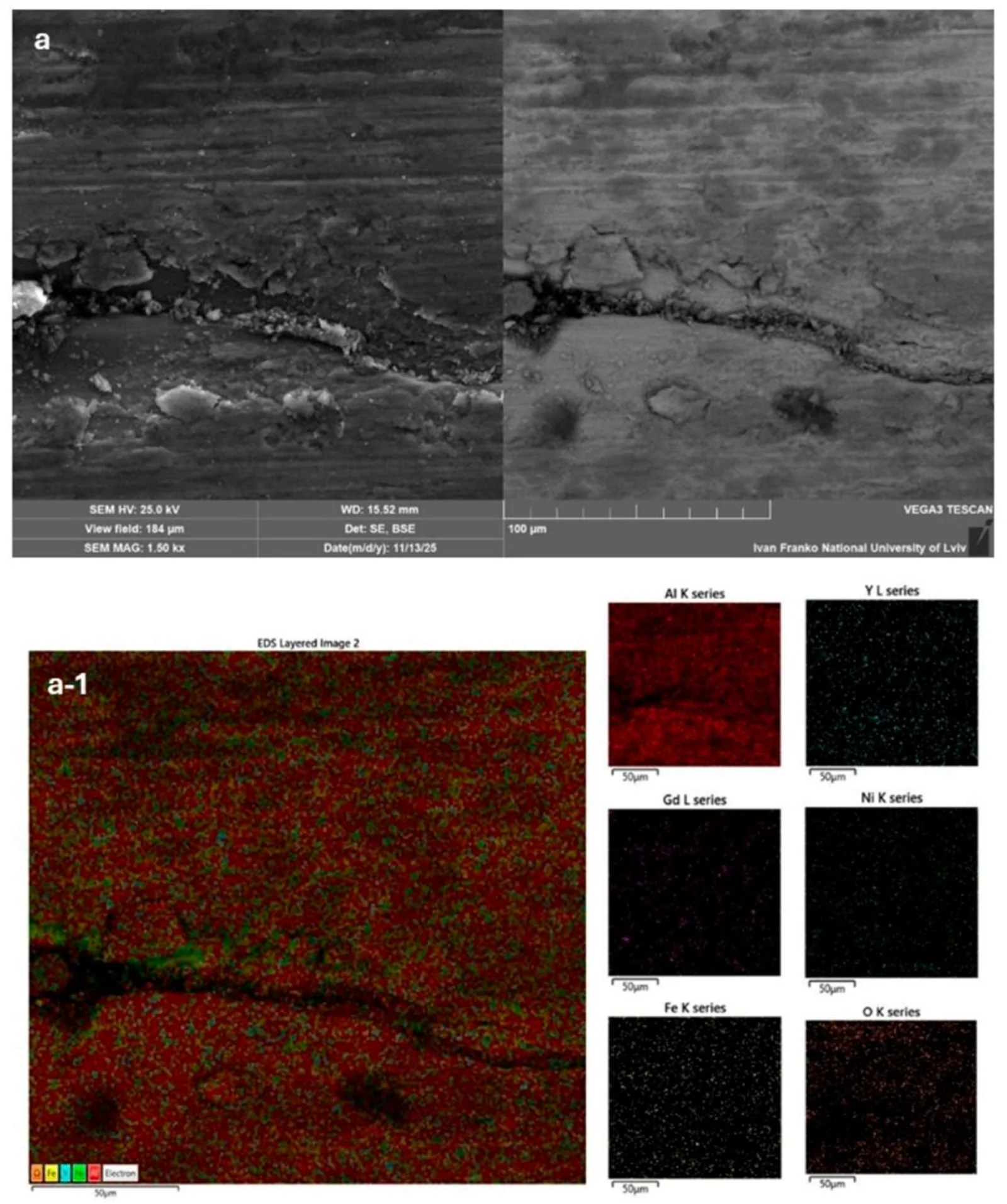

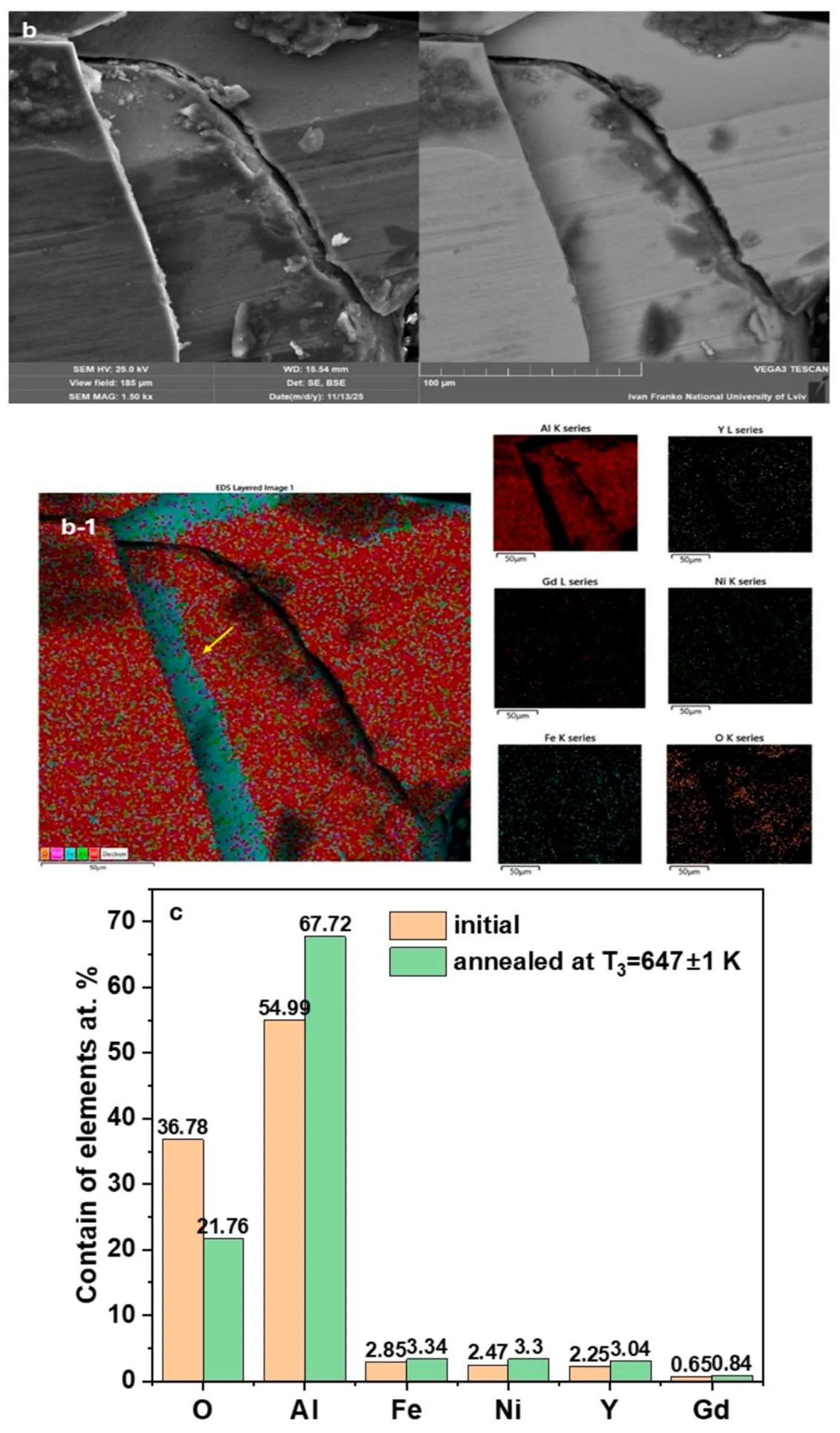
SEM images and EDS analysis of (**a**,**a-1**) the as-cast sample and (**b**,**b-1**) the sample annealed at T = 647 ± 1 K for 5 min following tribological testing. The yellow arrow indicates iron-enriched zone; (**c**) Dependence of the change in elemental composition (at.%) on the surface of the AMA following heat treatment.

SEM images and EDS analysis of the samples after tribological testing (Figure 6) show that annealing at 647 ± 1 K for 5 min leads to pronounced changes in the surface morphology, including the formation of cracks and local regions enriched in Fe and Gd (Figure 6b,b-1,c). As previously established [27], annealing of Al_87_Y_4_Gd_1_Ni_4_Fe_4_ under the same conditions leads to the formation of Al(Gd,Ni,Y,Fe), AlFe_2_Ni and GdFe_2_ phases. The formation of these phases may locally modify the oxidation behavior and mechanical stability of the surface layer, which could contribute to the pronounced improvement in wear resistance observed after annealing.

Quantitative EDS analysis was performed in three representative surface regions for each sample state (n = 3). The mean ± SD values (at.%) for the as-cast/annealed states were: O 36.78 ± 0.74/21.76 ± 0.44, Al 54.99 ± 1.10/67.72 ± 1.35, Fe 2.85 ± 0.06/3.34 ± 0.07, Ni 2.47 ± 0.05/3.30 ± 0.07, Y 2.25 ± 0.05/3.04 ± 0.06, and Gd 0.65 ± 0.01/0.84 ± 0.02; the relative SD did not exceed 2%. These EDS values are used to assess relative elemental redistribution.

### 4.4. Electrochemical Measurement of Al_87_Y_4_Gd_1_Ni_4_Fe_4_ Amorphous Alloys in the As-Cast State and After Annealing for 5 min at T_3_ = 647 ± 1 K in 0.3% NaCl

In our previous work [1], we presented a schematic representation of the surface oxide film formed on the amorphous alloy in a neutral environment. The electrochemical behavior of the alloy is strongly influenced by both surface topography and local chemical heterogeneity. Although the amorphous structure lacks conventional grain boundaries, which often act as preferential corrosion sites in crystalline materials, surface roughness, local compositional variations, micropores, and defects may provide pathways for electrolyte penetration. Chloride ions can adsorb at defective regions of the oxide film and penetrate through pores and microcracks, promoting local destabilization of the protective layer. Their interaction with the Al-rich oxide may facilitate local dissolution of Al-containing species and initiate active corrosion sites. At the atomic scale, this process can be associated with local disruption of Al–O bonds and enhanced ionic transport through structurally defective regions of the surface film [1,7,27].

**Figure 7:**
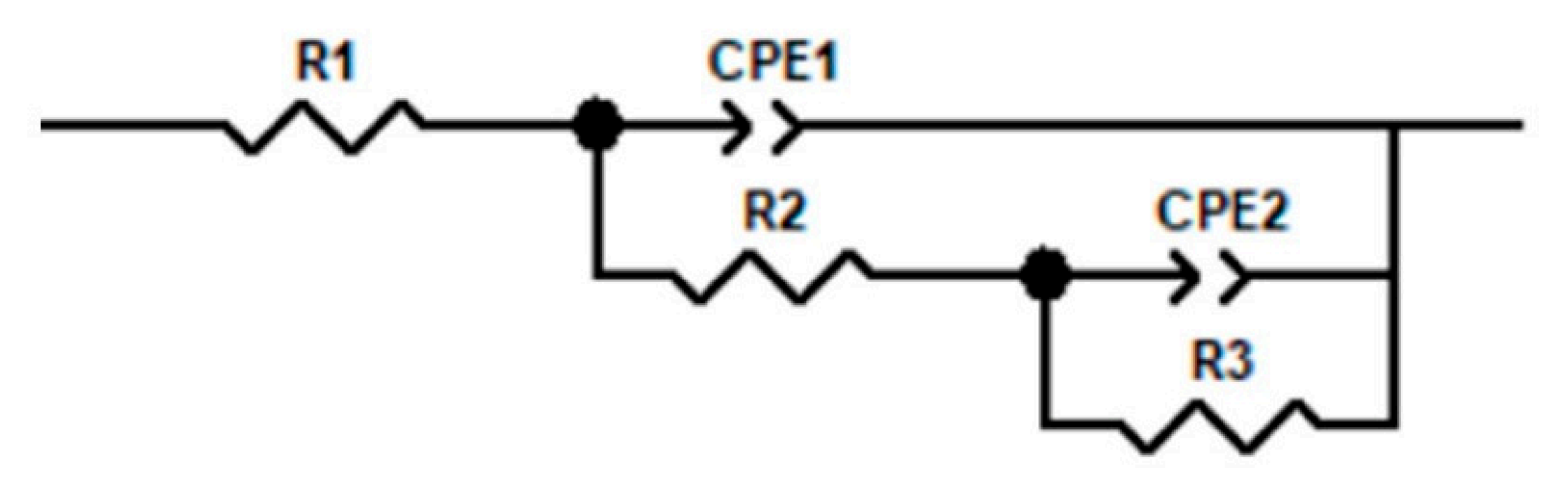
Equivalent electrical circuit used for fitting the EIS data of the amorphous Al_87_Y_4_Gd_1_Ni_4_Fe_4_ alloy in 0.3% NaCl solution [1,27].

Potentiodynamic polarization measurements complemented the EIS analysis. The corrosion potential (E_corr) and corrosion current density (i_corr) are summarized in Table 5. For each cycle number, the values obtained in the three independent experimental series were averaged. After the initial cycle, the annealed alloy showed more negative E_corr values and higher i_corr values in cycles 2–5, consistent with reduced electrochemical protection. The equivalent electrical circuit shown in Figure 7 was used to fit the EIS data.

**Figure 8:**
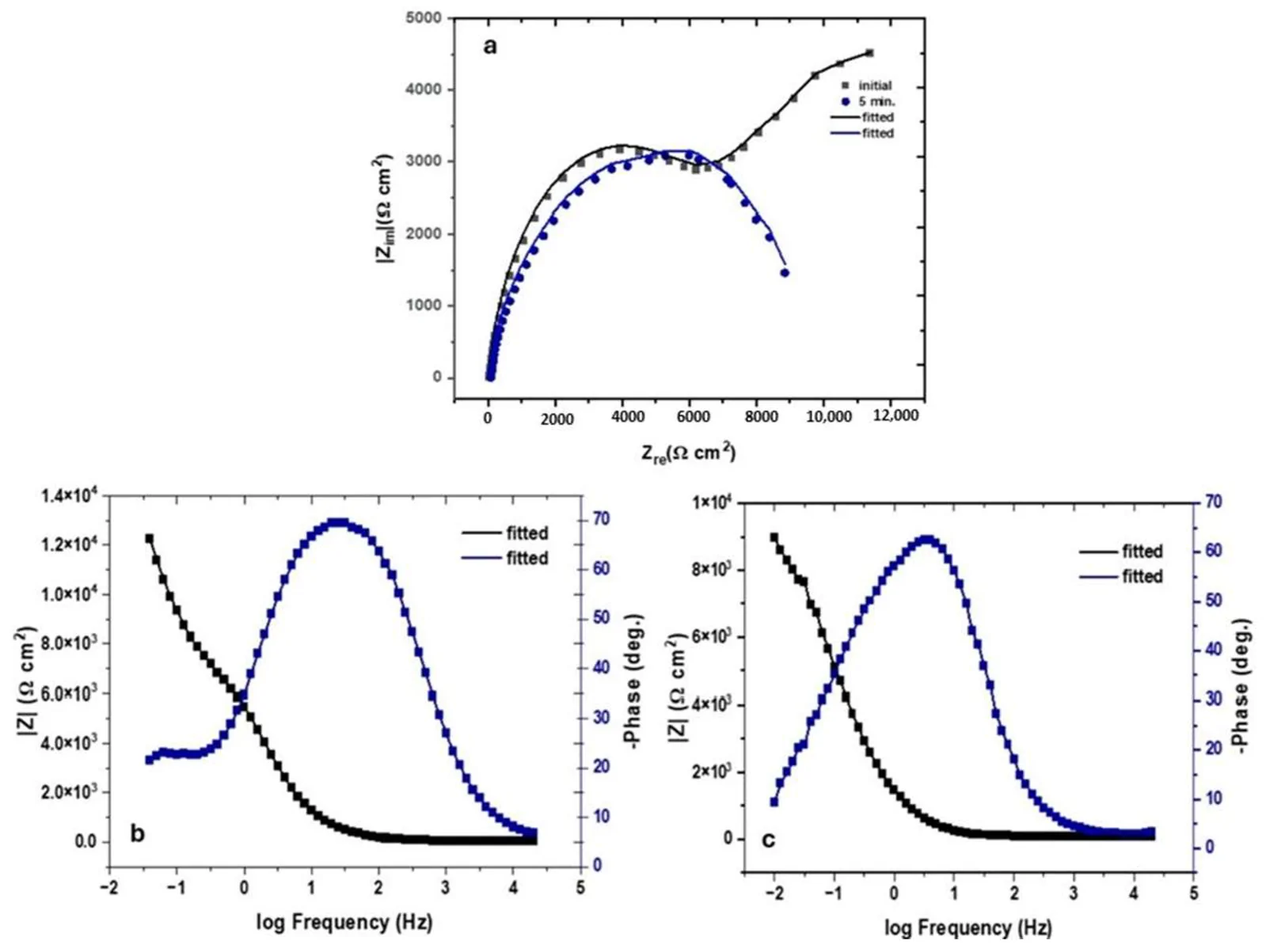
(**a**) Nyquist plots of the as-cast and annealed Al_87_Y_4_Gd_1_Ni_4_Fe_4_ amorphous alloy in 0.3% NaCl solution. Experimental data (symbols) and fitted curves (lines) obtained using the equivalent circuit shown in Figure 7. (**b**,**c**) Bode plots of impedance magnitude and phase angle for (**b**) the as-cast sample and (**c**) the sample annealed at 647 ± 1 K for 5 min.

The EIS results (Figure 8, Table 6) reveal pronounced changes in the electrochemical response of the Al_87_Y_4_Gd_1_Ni_4_Fe_4_ amorphous alloy after annealing at 647 ± 1 K for 5 min. The passive-film resistance, Rf, decreases from 7845 to 4335 Ω·cm^2^, indicating a reduction in the barrier properties of the surface film after heat treatment. At the same time, the CPEf-T parameter increases markedly from 2.15 × 10^−5^ to 12.2 × 10^−5^, whereas nf remains almost unchanged, decreasing only slightly from 0.86 to 0.85. The pronounced increase in CPEf-T together with the decrease in Rf indicates a substantial modification of the electrochemical response of the surface film and is consistent with the formation of a less effective protective barrier after annealing. This interpretation is also consistent with the changes in surface chemical state and heterogeneity revealed by XPS and SKP.

Significant changes are also observed in the electrochemical response of the electrode/electrolyte interface. The CPEdl-T parameter decreases from 40.3 × 10^−5^ to 29.9 × 10^−5^, while the exponent ndl decreases from 0.91 to 0.74. The pronounced decrease in ndl indicates a stronger deviation from ideal capacitive behavior and an increase in the heterogeneity of the electrode/electrolyte interface after annealing. This behavior may be associated with local disruption of the continuity of the passive film and with structural and compositional changes occurring during heat treatment.

The charge-transfer resistance, R_ct_, decreases from 8692 to 5262 Ω·cm^2^, further indicating a reduction in the resistance of the surface to electrochemical dissolution. Thus, although XPS shows an increase in the relative contribution of oxidized Al after annealing, the EIS parameters demonstrate that the resulting surface layer provides a less effective electrochemical barrier. The simultaneous decrease in Rf and Rct, together with the pronounced reduction in ndl, indicates that short-term annealing increases the electrochemical heterogeneity of the surface and facilitates charge-transfer processes at locally less protective regions of the oxide film. Such local degradation may enhance electrolyte penetration through defective regions of the passive layer and promote localized corrosion, including pitting.

From an engineering perspective, short-term annealing at 647 ± 1 K for 5 min may be advantageous for applications in which resistance to dry-sliding wear is the primary requirement and exposure to corrosive electrolytes is limited. In contrast, for components operating under continuous wet conditions or in chloride-containing environments, the decrease in R_f and R_ct, together with the polarization response, indicates reduced electrochemical protection; therefore, this heat-treatment condition should be used with caution or combined with additional surface protection. Because the tribological and electrochemical tests were performed independently under controlled laboratory conditions, these observations define the potential application range of the treatment rather than predict long-term service performance.

## 5. Conclusions

1. Short-term annealing of the Al_87_Y_4_Gd_1_Ni_8_ and Al_87_Y_4_Gd_1_Ni_4_Fe_4_ amorphous alloys for 5 min at 611 ± 1 K and 647 ± 1 K, respectively, produced pronounced changes in their surface electronic states. Heat treatment shifted the mean *CPD* towards more positive values and increased the distance over which the *CPD* values remained spatially correlated in both alloys. The effect of annealing on the magnitude of the local *CPD* variations, quantified by the *CPD*_rms_ parameter, depended on the alloy composition and was particularly pronounced for Al_87_Y_4_Gd_1_Ni_8_.
2. Annealing causes only minor changes in the overall surface topography. The roughness parameters increase slightly from R_a_ = 1.29 µm, R_q_ = 1.95 µm, and R_z_ = 9.52 µm in the as-cast state to R_a_ = 1.33 µm, R_q_ = 2.01 µm, and R_z_ = 9.92 µm after heat treatment.
3. Under the applied dry-sliding conditions, annealing improves wear performance: the specific wear rate decreases from 1.917 × 10^−3^ to 1.234 × 10^−3^ mm^3^/(N·m) (approximately 36%), the wear-scar width from 973 to 728 μm, and the maximum wear-track depth from 35.8 to 8.55 μm. SEM/EDS also reveals changes in wear-track morphology and local Fe/Gd enrichment.
4. XPS analysis demonstrates a redistribution of the surface chemical states rather than a simple increase in the total oxygen content. The relative contribution of oxidized Al increases from approximately 84.4% to 92.9%, while the contribution of non-oxide Al species decreases from approximately 15.6% to 7.1%. At the same time, yttrium becomes enriched in the near-surface region, whereas the relative proportions of the Y_2_O_3_-related and Y–O–Al components remain nearly unchanged at approximately 60% and 40%, respectively.
5. Despite the higher fraction of oxidized Al, annealing reduces electrochemical protection: R_f decreases from 7845 to 4335 Ω·cm^2^, R_ct from 8692 to 5262 Ω·cm^2^, and n_dl from 0.91 to 0.74; the polarization data show the same tendency after stabilization. These opposite functional responses are associated with the same annealing-induced surface reorganization. The formation of larger spatially correlated CPD domains, near-surface chemical redistribution, and nanophase formation may contribute to improved mechanical stability, while the accompanying increase in electrochemical heterogeneity may locally weaken the passive barrier. Thus, the treatment improves wear performance but reduces electrochemical protection in chloride solution.

## Figures and Tables

**Table 1:** Statistical parameters calculated from the *CPD* maps of the Al_87_Y_4_Gd_1_Ni_8_ and Al_87_Y_4_Gd_1_Ni_4_Fe_4_ amorphous alloys in the as-cast and annealed states. *CPD*_av_—arithmetic mean, *CPD*_rms_—root mean square deviation, *CPD*_sk_—skewness, *CPD*_ku_—excess kurtosis, and *CPD*_al_—autocorrelation length.

Sample (AMA)	*CPD*_av_ [V]	*CPD*_rms_ [mV]	*CPD*_sk_	*CPD*_ku_	*CPD*_al_ [µm]
Figure 1a (Al_87_Y_4_Gd_1_Ni_8_ as-cast)	−1.193	17.982	−0.248	0.944	163.196
Figure 1b (Al_87_Y_4_Gd_1_Ni_8_ 611 ± 1 K)	−0.813	38.974	0.005	0.198	211.877
Figure 2a (Al_87_Y_4_Gd_1_Ni_4_Fe_4_ as-cast)	−0.978	16.327	0.470	3.750	113.624
Figure 2b (Al_87_Y_4_Gd_1_Ni_4_Fe_4_ 647 ± 1 K)	−0.285	21.005	−0.448	1.107	271.798

**Table 2:** Elemental atomic concentrations and ratios for initial and annealed samples.

AMA	Atomic Concentration					Relative Ratio of Atomic Concentration		
	Al	O	Y	Ni	C	O/Al	O/Y	Al/Y
INITIAL	16.79	38.58	0.36	0.05	44.22	2.30	107.2	46.6
ANNEALED	16.60	33.04	0.53	0.00	49.83	1.99	62.3	31.3

**Table 3:** Surface chemical states of aluminum and yttrium with their corresponding binding energies (Al 2p and Y 3d_5/2_) and relative percentages obtained from peak deconvolution.

Sample	Element	Binding Energy (Al 2p/Y 3d_5/2_) [eV]	Relative Contribution [%]	Chemical State
Initial	Al 2p	71.37	8.56	Al^0^ (state 1)
	Al 2p	71.83	7.04	Al^0^ (state 2)
	Al 2p	74.20	84.40	Al_2_O_3_
Annealed	Al 2p	71.57	4.94	Al^0^ (state 1)
	Al 2p	72.10	2.20	Al^0^ (state 2)
	Al 2p	74.16	92.86	Al_2_O_3_
Initial	Y 3d_5/2_	156.88	59.9	Y_2_O_3_
	Y 3d_5/2_	158.93	40.1	Y-O-Al
Annealed	Y 3d_5/2_	157.00	60.0	Y_2_O_3_
	Y 3d_5/2_	159.05	40.0	Y-O-Al

**Table 4:** Surface roughness parameters (R_a_, R_q_, R_z_) of the amorphous Al_87_Y_4_Gd_1_Ni_4_Fe_4_ alloy after annealing for 5 min at T = 647 ± 1 K.

Roughness, μm	Time of Heat Treatment, min.	
	As Cast	5
R_a_	1.29	1.33
R_q_	1.95	2.01
R_z_	9.52	9.92

R_a_—arithmetic mean of the profile ordinate; R_q_—the average square of the profile; R_z_—the highest high profile.

**Table 5:** Corrosion potential (E_corr) and corrosion current density (i_corr) of the as-cast and 5 min annealed Al_87_Y_4_Gd_1_Ni_4_Fe_4_ alloy in 0.3% NaCl. Values represent the means of three independent experimental series (n = 3) for each consecutive polarization cycle; the relative SD did not exceed 2%. The five cycles were repeated measurements within each experimental series and were not treated as independent replicates.

Cycle	As-Cast E_Corr, V	As-Cast i_Corr, mA·cm^−2^	5 min E_Corr, V	5 min i_Corr, mA·cm^−2^
1	−1.35	0.575	−1.25	0.002
2	−1.24	0.741	−1.30	0.977
3	−1.18	0.986	−1.22	2.138
4	−1.16	0.871	−1.19	1.072
5	−1.15	0.794	−1.20	3.311

**Table 6:** Impedance parameters of the amorphous Al_87_Y_4_Gd_1_Ni_4_Fe_4_ alloy in 0.3% NaCl solution obtained from equivalent circuit fitting (Figure 7) as-cast and after 5 min of annealing at 647 ± 1 K.

Heat Treatment Time, min.	R_s_, Ω cm^2^	R_f_, Ω cm^2^	CPE-f-T (Ω^−1^ cm^−2^)×10^−5^	n_f	R_ct_, Ω cm^2^	CPE-dl-T (Ω^−1^ cm^−2^)×10^−5^	n_dl
as cast	43.01	7845	2.15	0.86	8692	40.3	0.91
5	86.55	4335	12.2	0.85	5262	29.9	0.74

## Data Availability

The original contributions presented in this study are included in the article. Further inquiries can be directed to the corresponding author.

## Abbreviations

The following abbreviations are used in this manuscript:

AMAs	Amorphous metal alloys
DSC	Differential Scanning Calorimetry
SKP	Scanning Kelvin Probe
CPD	Contact potential difference
XPS	X-ray photoelectron spectroscopy
EDS	Energy-Dispersive X-Ray Spectroscopy
